# Phytochemical Investigation and Reproductive Capacity of the Bulgarian Endemic Plant Species *Marrubium friwaldskyanum* Boiss. (Lamiaceae)

**DOI:** 10.3390/plants11010114

**Published:** 2021-12-30

**Authors:** Valtcho D. Zheljazkov, Ivanka B. Semerdjieva, Jan F. Stevens, Wenbin Wu, Charles L. Cantrell, Elina Yankova-Tsvetkova, Lyubka H. Koleva-Valkova, Albena Stoyanova, Tess Astatkie

**Affiliations:** 1 Department of Crop and Soil Science, Oregon State University, 3050 SW Campus Way, 109 Crop Science Building, Corvallis, OR 97331, USA; 2 Department of Botany and Agrometeorology, Agricultural University, Mendeleev 12, 4000 Plovdiv, Bulgaria; v_semerdjieva@abv.bg; 3 Department of Plant and Fungal Diversity and Resources, Institute of Biodiversity and Ecosystem Research, Bulgarian Academy of Sciences, 1113 Sofia, Bulgaria; e_jankova@abv.bg; 4 Department of Pharmaceutical Sciences and the Linus Pauling Institute, Linus Pauling Science Center 435, Oregon State University, SW Campus Way, Corvallis, OR 97331, USA; fred.stevens@oregonstate.edu (J.F.S.); wbwu318@hotmail.com (W.W.); 5 National Center for Natural Products Research, Agricultural Research Service, United States Department of Agriculture, University, MS 38677, USA; charles.cantrell@usda.gov; 6 Department of Plant Physiology, Biochemistry and Genetics, Agricultural University, Mendeleev 12, 4000 Plovdiv, Bulgaria; l_koleva2001@yahoo.com; 7 Department of Tobacco, Sugar, Vegetable and Essential Oils, Perfumery and Cosmetics, University of Food Technologies, 26 Maritza, 4002 Plovdiv, Bulgaria; aastst@abv.bg; 8 Department of Engineering, Faculty of Agriculture, Dalhousie University, Truro, NS B2N 5E3, Canada; astatkie@dal.ca

**Keywords:** Bulgarian endemic, phytochemistry, trichomes, SEM, nutlets, embryology

## Abstract

*Marrubium friwaldskyanum* Boiss (Lamiaceae) is a Bulgarian endemic species. Overall, the essential oil (EO) composition of *M. friwaldskyanum* was different from that of the other *Marrubium* species reported in the literature. The main EO constituents of *M. friwaldskyanum* were (*E*)-caryophyllene, germacrene D, and caryophyllene oxide. The effect of the harvest stage was significant only on α-copaene, (*E*)-caryophyllene, caryophyllene oxide, and τ-muurolol. The concentration of α-copaene (1.26–1.83% range of the total oil), (*E*)-caryophyllene (31–41%), caryophyllene oxide (6.4–11.8%), and τ-muurolol (1.3–2.8%) were the highest at 2–3 pair of leaves or before flowering and lower at flowering. The harvest stage did not significantly affect the concentrations of the other six identified EO compounds β-bourbonene (1.1%), α-humulene (2.8%), germacrene D (23.3%), bicyclogermacrene (2.85%), δ-cadinene (1.1%), and spathulenol (2.8%). In a separate experiment, grinding of the biomass prior to EO extraction had a significant effect only on the concentrations of D-limonene (0.24–3.3%) and bicyclogermacrene (3.6–9.1%). Grinding in water or without water, maceration, and addition of Tween^®^20 had rather small effects on the EO profile. The identified EO constituents and their mean concentrations in this experiment were (*E*)-caryophyllene (25.4%), germacrene D (17.6%), caryophyllene oxide (9.1%), spathulenol (6.5%), τ-muurolol (5.0%), carvacrol (3.9%), α-copaene (2.5%), β-bourbonene (2.5%), δ-cadinene (2.4%), α-humulene (1.8%), and Z-β-farnesene (1.3%). Embryological studies observed anther and the development of the male gametophyte and ovule and development of the female gametophyte of *M. friwaldskyanum*. Furthermore, pollen and seed viability assays were conducted, and mass spectrometry-based metabolomics analysis of an extract from shoots revealed the presence of 45 natural products, identified as flavonoids, phenolic acids, and (tri)terpenoids. Overall, the phytochemistry and some of the microscopic analyses distinguished this endemic species from other species in *Marrubium*.

## 1. Introduction

Plant chemicals are major sources for natural products (NPs) utilized in various industries such as pharmaceutical, food and beverage, and cosmetics. Natural products have a long history of clinical use and are a rich source of bioactive compounds [1]. The use of NP for the development of pharmaceutical products has increased significantly in recent decades [2,3]. Endemic plants represent untapped resources with potential as a source of NPs with new chemistry and utilization. Endemic plants have a restricted range of distribution, specific to the flora of a particular region or country. In most cases these plants are protected by local and international laws and their populations may be scarce and fragile and sometimes in remote and difficult to access areas. For these and other reasons, endemic plants are generally not well studied. However, again, they may have important chemical constituents or profiles with interest to various industries.

*Marrubium friwaldskyanum* Boiss. (Lamiaceae) is a Bulgarian endemic species and it has a very limited distribution. The species is under the protection of the Bulgarian biodiversity law with national conservation status [4], and it is considered critically endangered (CR). Critically endangered species are included in the Red Data Book of the Republic of Bulgaria [5]. Habitats of these species in Bulgaria are within the protected areas of the European ecological network Natura 2000. *Marrubium friwaldskyanum* is a herbaceous, perennial plant. Its stem is 20–60 cm high, not branched, with numerous hairs. The leaves are simple, with short petioles, 1.2–5.5 cm long, 0.7–3.4 cm wide, round or elliptical, uniformly toothed. The flowers are two-lipped, pale yellow, clustered (vertebrae) with a 6–9 mm long corolla, and elliptical, triangular, glabrous nuts 1.2–1.8 mm long. The species blooms from May to June, and forms fruits (seeds) from July to August. The species is distributed in grassy, shrub-dominated communities, on shallow, dry soils, more commonly on limestone in the middle, western Rhodopes and the Thracian Plain in Bulgaria [5]. Its habitats are characterized by high temperatures and low levels of precipitation [6]. There are no previous studies on the phytochemical composition and the reproductive potential of *M. friwaldskyanum.* The limited distribution of this species might be due to issues with its reproductive potential.

The endemic *M. friwaldskyanum* has similar morphological traits with those of *Marrubium vulgare* (horehound), which was the number one top-selling herbal supplements in 2018 for the U.S. mainstream multi-outlet channel [7]. *Marrubium vulgare* L. fetched USD 146,624,255 in 2018 in the U.S. Mainstream Multi-Outlet Channel alone [7]. We could speculate that a similar but endemic species that grows high in the mountains of Bulgaria on very poor soils could also have such success if introduced into cultivation. This emphasizes the important practical aspects of research on endemic species. The aims of this study were (1) to reveal reproductive capacity that may be contributing to the limited distribution of *M. friwaldskyanum* and (2) to characterize its phytochemicals composition. The hypotheses of this study were that (1) the EO composition of Bulgarian endemic species will have a unique and different profile from the EO profile of related species in the same family, and (2) it may have issues with its reproductive capacity explaining its endemicity. This is the first report on the endemic species *M. friwaldskyanum* phytochemistry, embryology, and surface analyses of nutlet (seed) and pollen structure and seed viability.

## 2. Results

### 2.1. Essential Oils Analysis

#### Qualitative Composition of the Essential Oil (EO)

The major EO constituents of *M. friwaldskyanum* were (*E*)-caryophyllene, germacrene D, and caryophyllene oxide. As indicated in the statistical analyses, the effect of harvest stage was significant only on α-copaene, (*E*)-caryophyllene, caryophyllene oxide, and τ-muurolol (Table 1). The concentration of α-copaene (1.26–1.83% range of the total oil), (*E*)-caryophyllene (30.8–41.0%), caryophyllene oxide (6.35–11.8%), and τ-muurolol (1.3–2.8%) were the highest at 2–3 pair of leaves or before flowering and lower at flowering (Table 1). The harvest stage did not significantly affect the concentrations of the identified other 6 EO compounds β-bourbonene (1.1%), α-humulene (2.8%), germacrene D (23.3%), bicyclogermacrene (2.85%), δ-cadinene (1.07%), and spathulenol (2.83%) (Table 2).

Grinding of the biomass prior to EO extraction had a significant effect only on the concentrations of D-limonene (0.24–3.25%) and bicyclogermacrene (3.55–9.14%) (Table 3). The concentration of D-limonene was the highest in the grinding of the biomass in water and in the treatment with Tween^®^20 followed by immediate distillation, and lower in the whole plant distillation. Grinding with water without Tween^®^ treatment reduced the concentration of D-limonene, while grinding without water apparently resulted in the loss of D-limonene (Table 3). The concentration of bicyclogermacrene in the EO was higher at whole plant distillation and lower at the fresh/ground with water treatment; the other treatments were not significantly different (Table 3).

Grinding of the biomass prior to distillation did not affect the concentrations of (*E*)-caryophyllene (25.4%), germacrene D (17.6%), caryophyllene oxide (9.1%), spathulenol (6.5%), τ-muurolol (5.0%), carvacrol (3.9%), α-copaene (2.5%), β-bourbonene (2.5%), δ-cadinene (2.4%), α-humulene (1.8%), Z-β-farnesene (1.3%), and α-pinene (0.3%).

### 2.2. Phytochemical Determination

Screening of the extract of the aboveground plant parts against the Enzo Life Sciences Natural Products Library revealed the presence of 45 compounds that were identified as flavonoids [compound names followed by their % ion abundance of total ion current of the 45 compounds] (apigenin [1.1%], apigenin-7-*O*-glucoside [0.6%], diosmetin [<0.1%], eriocitrin [<0.1%], eriodictyol [0.3%], eriodictyol-7-*O*-glucoside [0.1%], daidzein [<0.1%], dihydrorobinetin [<0.1%], diosmin [<0.1%], formononetin [<0.1%], hesperitin [<0.1%], isoquercetrin [17.9%], isorhamnetin [0.3%], isorhamnetin-3-*O*-glucoside [6.0%], isorhamnetin-3-*O*-rutinoside [22.1%], isorhoifolin [<0.1%], kaempferol-7-neohesperidoside [<0.1%], luteolin [0.3%], marein [0.1%], maritimein [2.1%], naringenin [1.0%], naringenin-7-*O*-rutinoside [<0.1%], pratol [<0.1%], rhoifolin [0.6%], rutin [34.1%], syringetin-3-*O*-glucoside [<0.1%], flavokawin A [<0.1%], homobutein [<0.1%]), phenolic acids (caffeic acid [1.4%], chlorogenic acid [4.9%], ferulic acid [2.6%], rosmarinic acid [<0.1%]), anthraquinones (emodin [<0.1%]), terpenoids (asiatic acid [0.7%], betulinic acid [1.2%], oleanolic acid [1.2%], ursolic acid [1.2%], caryophyllene [<0.1%], lagochiline [0.4%], sclareol/peregrinol/vulgarol [<0.1%]), and other compounds (usnic acid, scopoletin, shikimic acid, phytosphingosine, vanillylacetone; all <0.1%).

### 2.3. Embryological Research

On the base of light microscopy observations, permanent microscopic slides were prepared from flower buds, and the main features of male and female generative spheres of *M. friwaldskyanum* were revealed.

#### 2.3.1. Anther and Development of the Male Gametophyte of *M. friwaldskyanum*

**Figure 1:**
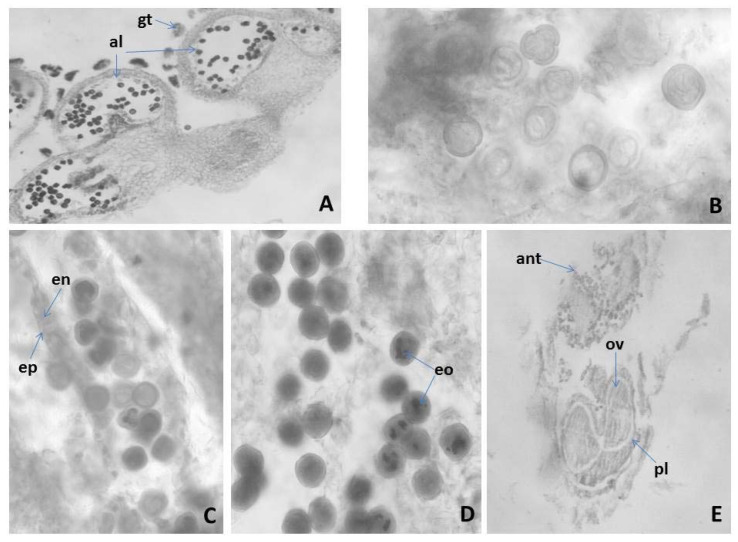
Anther and development of the male gametophyte revealed after light microscopy observations: (**A**) tetrasporangiate anther; (**B**) two celled mature pollen grains; (**C**) mature pollen grains and anther wall with epidermis and fibrous endothecium; (**D**) mature pollen grains; (**E**) flower with anther and ovary; al—anther locule, gt—glandular trihomes, ep—epidermis, en—endothecium, eo—essential oil, pl—pistil, ant—anther, ov—ovule Magnification: (**A**,**E**) (×100); (**B**–**D**) (×400).

The *M. friwaldskyanum* anther is four-locular (Figure 1A). The anther walls are four-layered, built of epidermis, endothecium, middle layer and tapetum of the secretory type. A characteristic element of the epidermal cells is the formation of glandular trichomes (Figure 1A) and of the entothecial cells, the development of fibrous thickenings after the formation of one-nucleated pollen (Figure 1C). The tapetum remains cellular up to the maturity of the anther. Formation of placentoids between anther locules was observed. Meiosis in microspore mother cells run normally with some insignificant deviations expressed in lagging behind chromosomes and chromosome bridges. After the simultaneous microsporogenesis, predominantly tetrahedral tetrads were formed. An indication of properly running meiosis was the presence of equal in size pollen grains at a later stage of development of most anthers (Figure 1B). The mature pollen grains are two celled (Figure 1B), and the accumulation of essential oil (EO) in the form of spherical inclusions was observed in them (Figure 1D).

#### 2.3.2. Ovule and Development of the Female Gametophyte

**Figure 2:**
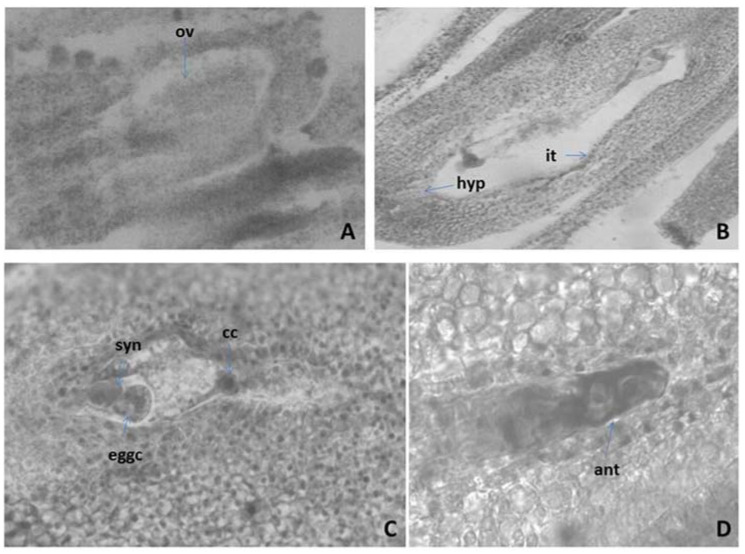
Ovule and development of the female gametophyte revealed after light microscopy observations: (**A**) Anatropous unitegmic ovule; (**B**) Mature *Polygonum*-type embryo sac (ES); (**C**) Mature ES with egg apparatus and central cell; (**D**) Antipodals in the ES—cavity ov—ovule, hyp—hypostase it—integumental tapetum (endothelium), eggc—egg cell, syn—synergids, cc—central cell, ant—antipodal. Magnification: (**A**,**B**) (×100); (**C**,**D**) (×400).

The pistil in *M. friwaldskyanum* is in the upper position (Figure 1E), 4-locular. In each loculus, an anatropous, tenuinucellate unitegmic ovule forms (Figure 2A). The development of the female gametophyte follows the *Polygonum* type (Figure 2B). The mature embryo sac consists of egg apparatus composed of an egg cell and two synergids with typical structure and position for these cells: pear-shaped egg cell with a nucleus at the chalazal, vacuole at the micropylar pole, and synergids similar in shape to the egg cell but with an opposite arrangement of nucleus and vacuole (Figure 2C); and three antipodals located at the chalazal part of the embryo sac (Figure 2D). From the specialized ovule structures in the studied species were established integumental tapetum from the innermost layer of the integument, and hypostase from cells of nucellus below the antipodals (Figure 2B). The embryo and endosperm are formed as a result of double fertilization. Embryogenesis runs according to the *Onagrad*-type. Apomixis was not registered.

#### 2.3.3. Pollen and Nutlets (Seed) Viability

**Figure 3:**
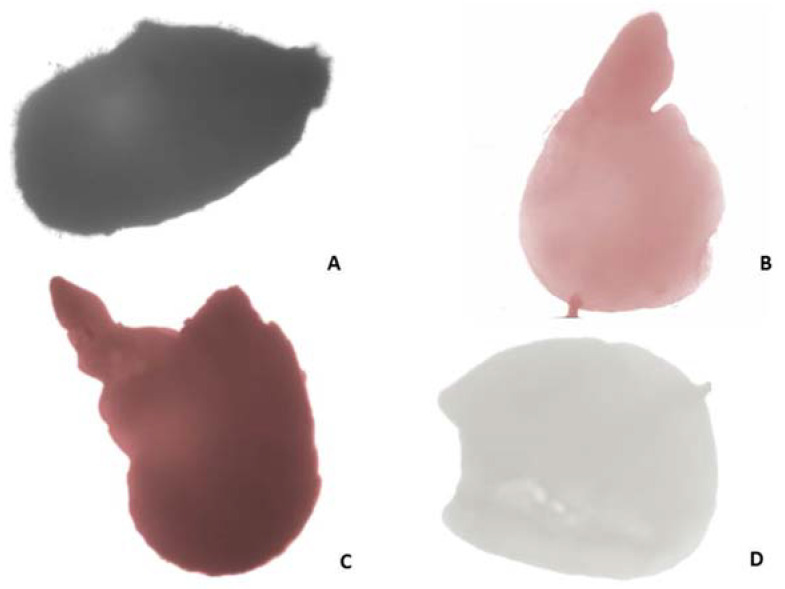
Estimation of seed viability according to Tetrazolium test: (**A**) Entire seed; (**B**,**C**) viable embryos: pink colored/B/, stained in dark red/C/; (**D**) unstained non-viable embryo.

**Figure 4:**
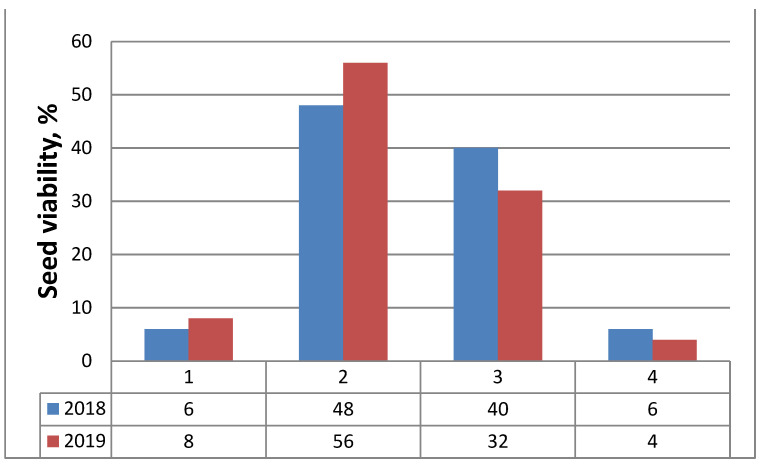
Frequency of seeds (embryos) viability (%) assessed by tetrazolium test in classes according to the color patterns of tested embryos for two consecutive years (2018, 2019): 1—Class I, viable embryos (stained in red); 2—Class II, viable embryos (pink colored embryos); 3—Class III, non-viable embryos (not stained); 4—Class IV, non-viable (empty) seeds.

After acetocarmine staining, the cytoplasm and nuclei of viable pollen grains were stained in red while non-viable, empty, and shrunken pollen grains remained unstained. The results of the study showed high viability of the mature pollen (83.36 ± 3.43% in the flower collected in 2018, and 82.96 ± 2.61% in the flower collected in 2019). On the basis of tetrazolium testing, the seeds (embryos) were differentiated into four classes (Figure 3): Class I—viable embryos (embryo stained in red-Figure 3B); Class II—viable embryos (pink-colored embryos-Figure 3C); Class III—non-viable embryos (not stained embryos -Figure 3D); Class IV—non-viable (empty) seeds. According to the criteria for interpretation of the tetrazolium test results given by Moore [8], the viable embryos are represented by the color patterns of Classes I and II. Thus, the resulting percentage of viable seeds was 54% for the seeds gathered in 2018, and 64% for seeds gathered in 2019 (Figure 4).

### 2.4. Seed (Nutlets) Germination

The results of germination energy (%) and germination (%) obtained from various light—emitting diodes (LED) treatments are shown in Table 4. The testing of *M. friwaldskyanum* seeds under light with different spectrum were innovative and not reported for this species. The germination energy varied from 24% (N) to 27% (W) (Table 4). The germination of seeds was 48–58% (Table 4).

### 2.5. Morphological Analysis by Scanning Electron Microscopy (SEM)

#### 2.5.1. Leaves, Stem, Calyx and Corolla Surfaces

**Figure 5:**
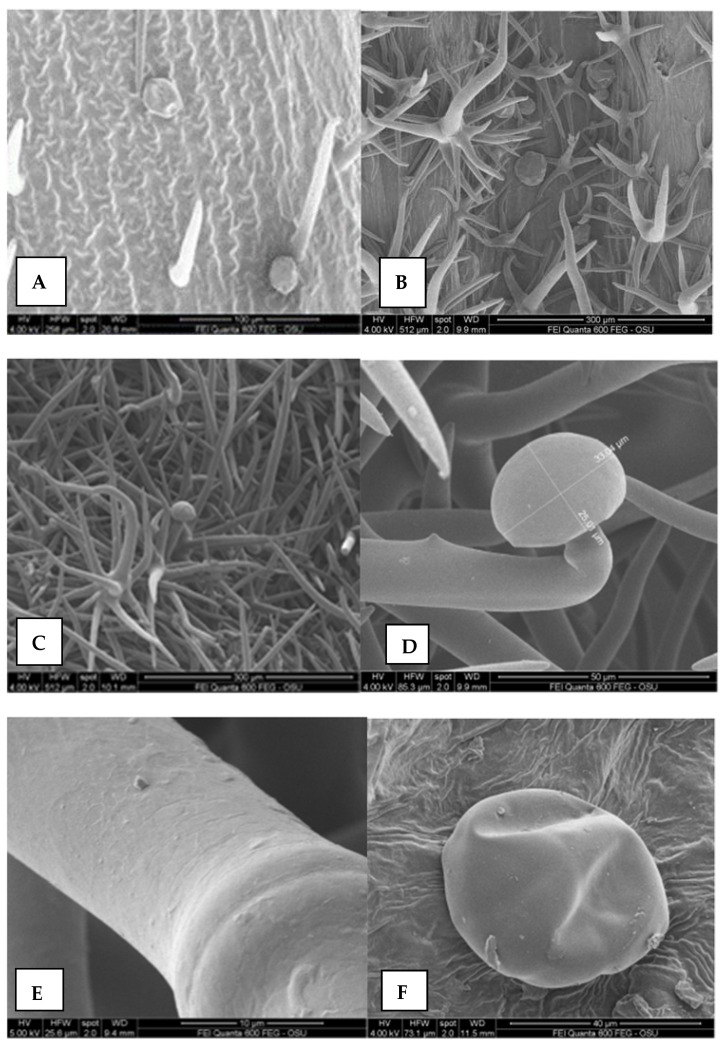

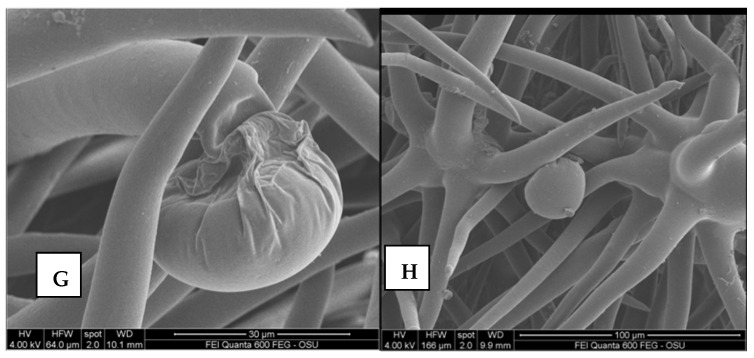
The leaves and calyx surfaces revealed after scanning electron microscopy (SEM) observations; (**A**) calyx inner surface with non-glandular trichomes; (**B**) calyx outer surface with non-glandular trichomes; (**C**) leaves surfaces with non-glandular and glandular trichomes; (**D**) close up of calix outer surfaces with non-glandular and glandular trichomes; (**E**) surfaces of non-glandular trichomes; (**F**) glandular peltate trichome, calyx; (**G**) glandular capitate trichome with some non-glandular trichomes; (**H**) leaves surfaces with non-glandular and glandular capitate trichomes.

The study found that all parts of the *M. friwaldskyanum* plant (leaves, stem, sepals, petals) were covered with non-glandular and glandular trichomes (Figure 5A–H). The observed non-glandular trichomes were of two types, namely: tree-like branched (Figure 5B,C,H) and unbranched (Figure 5A), and unicellular (Figure 5A) or bicellular (Figure 5B). The non-branched unicellular and bicellular trichomes were observed only on the inner surface of the calix (Figure 5A,B), the branched ones were observed on the surface of all plant parts. The tree-like branched trichomes were made up of unevenly long branches (Figure 5H). The branches varied in several respects, namely: (1) the number of cells that make them; (2) density; (3) position of the tip of the branches in space.

The tree-like trichomes were branched on the base and that gave them a star-shaped form (stellate). The main central stem of the trichome was the largest and consisted of either one or two cells. The two-cell branch had strongly convex, thickened internodes observed between the individual cells (Figure 5C,D). Trichomes are with two-cell central branches, with thick nodes between them (Figure 5B,C). The tips of some of the trichomes were straight, while in others they were curved like hooks (Figure 5B,C). The surface of the trichomes exhibited a network-like sculpture, with waxes that have an irregular plate-like shape (Figure 5F,H).

The glandular trichomes, where the terpenes are synthesized and accumulated were two main types: peltate and capitate (Figure 5A,C–E,G,H). The peltate glandular trichomes were composed of a stalk and a head and the stalk may be unicellular or multicellular (Figure 5D,F–H).

#### 2.5.2. Nutlets (Seeds) and Pollen Surfaces

##### Nutlets

**Figure 6:**
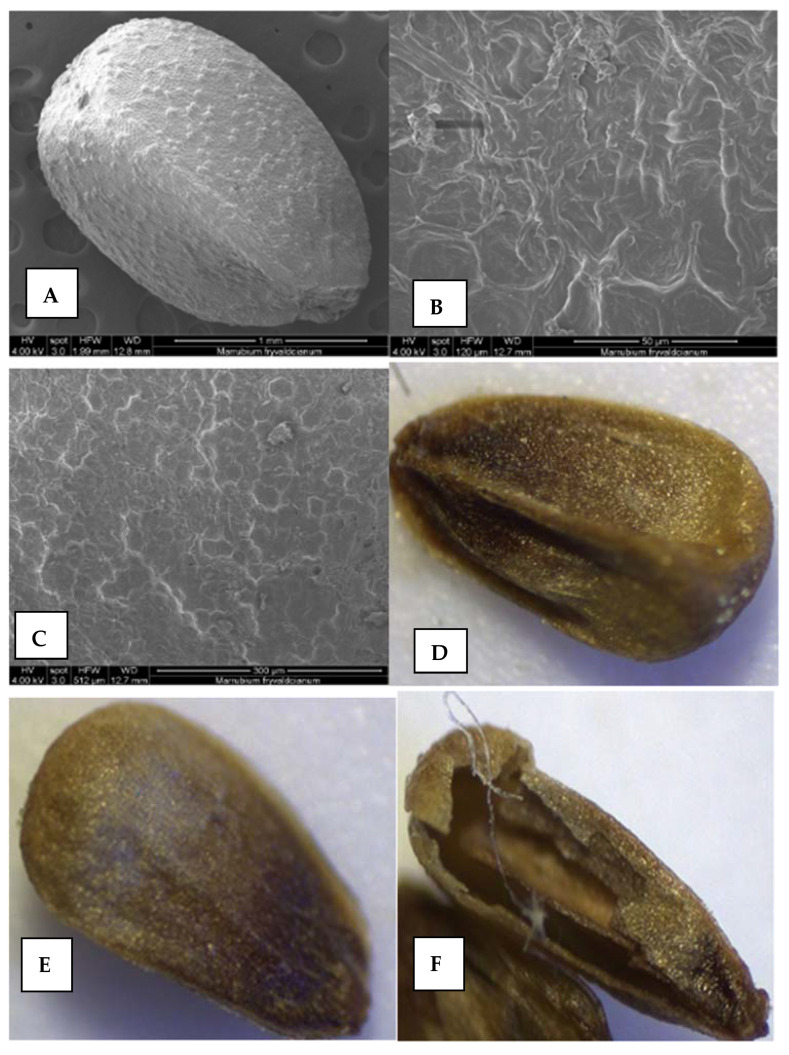

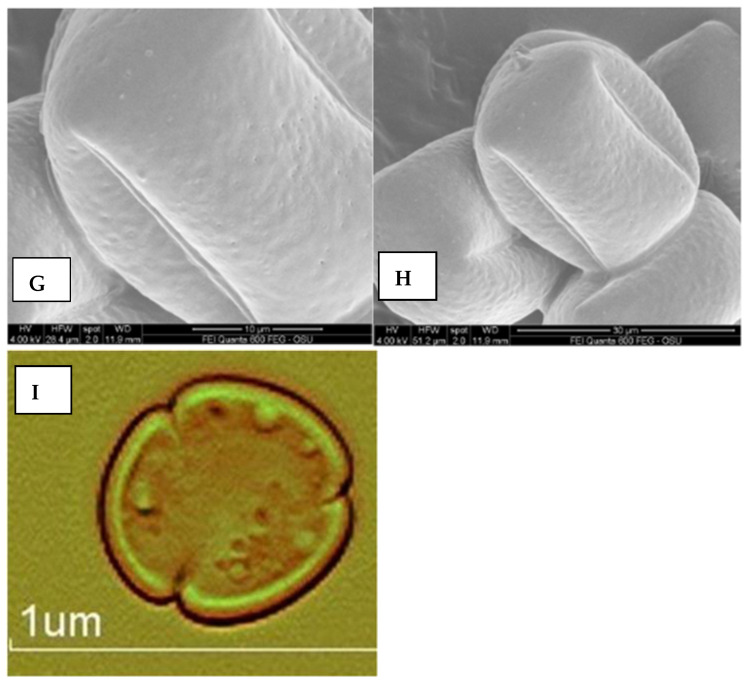
The nutlets and pollen surfaces revealed after scanning electron microscopy (SEM) and light microscopy (LM) observations. (**A**–**F**) nutlet; (**G**–**I**) pollen surfaces; (**I**) polar view.

The nutlets of *M. friwaldskyanum* are dark brown to black, 217 µm long and 132 µm wide. The nutlets have an ovoid oblong form, flattened at the apex, with clearly formed lateral winged edges (Figure 6A–F). The surface of the nutlet has specific wart formations (Figure 6A–C). According to the terminology of Barthlott and Ehler [9], the surface is from tabular type to concave type. The anticlinal and periclinal walls of the spermoderma form polygonal shapes (honeycomb-like cells). The periclinal walls of the spermoderma have slight striations, while the anticlinal walls are arcuately curved, with a triangularly pointed or thickened edge (Figure 6).

##### Pollen

The pollen in *M. friwaldskyanum* is prolate-spheroidal, tricoplate. An exine had a smooth surface (psilate). Mesocolpium was oval, and there is a small protuberance (papilla). The pores are scattered and are arranged randomly (Figure 6G–I).

## 3. Discussion

### 3.1. Essential Oils Analysis

This is the first report on the endemic species *M. friwaldskyanum* phytochemistry, embryology, surfaces analyses of nutlets (seed) and pollen structure, and seed viability.

Generally, the EO composition of *M. friwaldskyanum* was different from the EO profile of other *Marrubium* species. For example, Sarikurkcu et al. [10] examined another endemic species, *Marrubium parviflorum* subps. *oligodon* Boiss. and reported major EO constituents (Z, Z)-farnesyl acetone (19.3%), caryophyllene oxide (15.9%), and pulegone (7.2%). Mahmoud et al. [11] reported thymol (29.6–60.7%), carvacrol (0.5–19.3%), m-cymene (1.0–14.2%), γ-terpinene (1.1–12.1%), thymol methyl ether (0.4–10.4%) and α-himachalene (0.0–10.3%) as the principal components of *Marrubium vulgare* (common horehound). A study on *M. vulgare* natural populations from 10 areas in Algeria grouped the collected accessions into five chemotypes: β-bisabolene type (3.1–43.4%), δ-cadinene type (0.2–34.2%), (E)-β-farnesene type (1.4–34.8%), β-caryophyllene type (3.4–43.1%) germacrene-D type (2.1–37.9%) [12].

### 3.2. Essential Oil Yield

The EO yield in this study varied from 0.02% (% in absolute dry weight biomass) to 0.1%, which is comparable to the EO of some other *Marrubium* species. Generally, *Marrubium* species including the commonly used *M. vulgare* are known for their very low EO yield (content). The EO yield of a number of *Marrubium* species (with the exception of that of *M. vulgare*) was recently reviewed by Yabrir [13] and was found to vary from 0.01% in *M. parviflorum* Fisch. & C.A. Mey. subsp. *oligodon* (Boiss.) and from *M. aschersonii* Magnus [14] up to 0.8% in *M. propinquum* Fisch. and C.A. Mey hydrodistilled with Clevenger [15] and even up to 0.91% from *M. astracanicum* Jacq [16]. The EO yield values reported by the latter authors were surprisingly high and may be difficult to achieve.

### 3.3. Phytochemical Determination

Our phytochemical survey detected 48 natural products by LC-MS/MS matching with the Enzo Life Sciences Natural Products library consisting of 500 authentic standards. The advantage of library screening is that it is fast and does not require laborious isolation and de novo characterization of compounds. A disadvantage of the method is that characteristic compounds are missed if they do not occur in the library. Moreover, there is a risk of false-positive detection of compounds. For instance, preperegrinin has been reported for *M. friwaldskyanum* [17] but was not detected in our study. Indeed, the cited authors [17] did not provide details on the origin of the plant material and it is not clear if the authors indeed analyzed *M. friwaldskyanum*. However, preperegrinin is structurally related to the diterpene lagochilline, which we did detect but has not previously been reported for *Marrubium* species. Consistent with previous phytochemical reports for *Marrubium* species [18] we detected apigenin, luteolin, quercetin, kaempferol, isorhamnetin, either in their aglycone form or as their O-glycosides.

### 3.4. Embryological Research

Most of the established embryological features—tetrasporangiate, 4-layered anthers, tapetum of the secretory type, normal meiosis of the simultaneous type, anatropous tenuinucellate ovule, *Polygonum*-type development of female gametophyte are characteristics of other species of the Lamiaceae family [19,20]. There is additional evidence of the invariable embryological characteristics in this family, as was established previously by other researchers [21].

The observed glandular trichomes on the epidermis of the anthers were also described in *Agastache foeniculum* (Pursh) Kuntze [22], *Hyssopus officinalis* L. [23], *Salvia tomentosa* Mill. [24], and *Nepeta cataria* L. [25]. The absence or presence of glandular trichomes in different species could be used as a valuable diagnostic characteristic within the Lamiaceae [26]. The observed formation of placentoids in the anther locules of studied species is described as typical elements of the anthers of the representatives of Lamiaceae [19,20].

This study found that the EO seems to accumulate in mature pollen grains of *M. friwaldskyanum*. There have been similar reports on other species of Lamiaceae. The EO on pollen grains may play a role against abiotic stresses [27].

Another feature of the sample was the presence of hypostase, which is an organized tissue at the base of the nucellus and integument. In tenuinucelate ovules, such as those of the Lamiaceae family, hypostasis is located immediately below the embryo sac. It is believed that there is a relationship between the length of the hypostase and the pattern of nucellus, teguments, and the embryo sac. In the ovule, usually after the 2-, 4-nucleate embryo sac stage, a hypostase forms in its chalazal region. Figueiredo and Köhler [28] reported that during female gametogenesis the maternal tissues tightly regulate megagametophyte formation and the interplay between the sporophyte and the fertilization products, embryo and endosperm, has major implications in the formation of a viable seed [28].

The established high pollen viability is a prerequisite for successful pollination, and the normal running of the processes in the female generative sphere, of successful fertilization and embryo formation. The decrease in the resulting seed viability to 54% and 64% in different years can be explained by secondary variations in seed quality due to the influence of environmental factors. The mode of reproduction is of crucial importance in the reproductive strategy of each plant species and guarantees the successful regeneration of its populations. The revealed peculiarities of the structures and processes in the generative sphere of *M. friwaldskyanum* characterized it as a sexually reproducing species (no apomictic development was observed), which together with the normal formation of male and female gametophytes without deviations and degenerative processes, provides stability in the structure and size of the populations of the species. At the same time, the strict sexuality of the species limits its adaptive capabilities and is the probable cause of its attachment to certain environmental conditions that in turn cause its endemism. The differentiation in the level of seed viability in different years, estimated in the study, proved the dependence of this parameter on the climatic conditions in the year of seeds collection established, which is also reported in other species from Lamiaceae such as *Sideritis scardica* Griseb. [29].

### 3.5. Nutlets (Seed) Germination

Germination shows the seed’s ability to produce a normal seedling and under adverse conditions, an adaptive trait for survival in changing environmental conditions [30]. As mentioned in the introduction, *M. friwaldskyanum* has a limited distribution in Bulgaria. One of the reasons for the limited distribution of the species could be the low germination or a certain type of dormancy of the seeds [31]. Seed germination is influenced by several factors including air temperature, soil moisture, nutrients in the soil, and photoperiod [32]. This is the first report on germination tests on *M. friwaldskyanum* seeds.

There are many studies on the phytochemical benefits of growing various plant species under light-emitting diode (LED) exposure [32,33,34]. On the other hand, there are publications on *M. vulgare* (the most common and widely used *Marrubium* species) seed testing [35,36,37]. The results from this study showed that the highest Germination energy (27%) and Germination (58%) were obtained by irradiating the seeds with fluorescent white light. The different combinations of red/blue light obviously suppress the germination energy and germination of *M. friwaldskyanum* seeds. Similar results were obtained by Benvenuti et al. [36] for *M. vulgare*. The latter authors reported that far-red light inhibited seed germination in *M. vulgare* [36]. In summary, light affects the germination of seeds through phytochromes. Red light (600–700 nm) activates the phytochromes system and stimulates germination, while far-red light (700–800 nm) inactivates it and has an inhibitory effect on germination. Blue light inhibits germination and when combined with red can reduce its germination activating effect [38].

### 3.6. Morphological Analysis by Scanning Electron Microscopy (SEM)

#### 3.6.1. Leaves, Stem, Calyx, and Corolla Surfaces

The indumentum of plants, the seeds, and pollen surfaces are valuable taxonomic characteristics [39,40]. The morphological characteristics of leaf, indumentum, number and form of calyx teeth, and bracteole were important for describing the *Marrubium* species [41]. In this study, light and SEM microscopy analyses of the leaves, nutlets, and pollen of the endemic species *M. friwaldskyanum* were conducted and reported for the first time. We observed two types of trichomes (non-glandular and glandular), which are characteristics of Lamiaceae species [42,43,44]. The non-glandular trichomes (stellate and branched hairs) were densely distributed while the glandular (capitate and peltate) were scattered among them on fully expanded mature leaves of *M. friwaldskyanum*.

Most studies on *Marrubium* species were on species distributed in Turkey and Iran, as plants of the genus *Marrubium* are distributed in the Mediterranean region, Asia, and Africa [45,46]. The diversity found in the structure of non-glandular trichomes in this study was previously observed by Ahvazi et al. [47] in other species of the genus (*M. astracanicum* Jacq., *M. anisodon* K.Koch, *M. cordatum* Nábelek, *M. crassidens* Boiss, *M. duabense* Murata, *M. propinquum* Fisch. & C.A.Mey. and *M. vulgare*) distributed in Iran. The latter authors found that the main hair type in other *Marrubium* species was stellate with unequal rays, the general trichome shapes were similar but there were variations in stellate trichomes based on their long branch’s length, the short branch’s length, and the number of short branches. In this study, unbranched unicellular and bicellular hairs were found on the inner surface of the sepals, as well as a reticular sculpture of all hairs. This is a new finding; such observations on *Marrubium* species have not been reported previously.

The indumentum of Lamiaceae is a valuable taxonomic characteristic and glandular trichomes are important for secretions of EO [39,40,48,49]. The most important feature of the secretory tissues is the ability to synthesize, accumulate, and release the chemical substances of primary and secondary metabolism [50].

#### 3.6.2. Pollen Surfaces

According to Erdtmant [51], pollen morphology is an important indicator of systematic relationships between plants in Lamiaceae. Based on the pollen characteristics, Erdtmant [51] divided Lamiaceae into two groups: (1) two nuclear pollen, tricolpate, and (2) tree nuclear pollen, heterocolpate (four apertures or six apertures). In the first group, Erdtmant [51] put genus *Marrubium* but there is information for *Marrubium* species with tetracolpate and syncolpate pollen [45]. We observed tricolpate pollen in *M. friwaldskyanum*. Generally, the exine surfaces of pollen in the genus *Marrubium* can be quite variable. Akgül et al. [45] reported psilate-perforate (smooth) exine in *M. vulgare*, *M. anisodon* K.Koch, *M. parviflorum* Fisch. and C.A.Mey., etc, reticulate exine in *M. persicum* C.A.Mey., *M. globosum* Montbret & Aucher ex Benth., etc., ruguate-reticulate in *M. vanense* Hub.-Mor., and granulate-perforate in *M. depauperatum* Boiss. & Balansa while Firat [52] reported psilate-reticulate exine in *M. eriocephalum* Seybold.

#### 3.6.3. Nutlets Surfaces

The Schizocarpic fruit in the Lamiaceae species separates into four dry indehiscent nutlets at maturity [53]. As the pericarp of the nuts fuses with the seeds during the development, previous reports very often identified those to be the same thing. The surfaces and seeds morphology of *Marrubium* species distributed in Turkey were well documented [45,52]. The nutlets of *Marrubium* species may vary in size, color, form, and nutlet surface, and these traits may have systematic significance [45,52,54]. The nutlets morphology of *M. friwaldskyanum* in this study was observed and reported for the first time. In most previous reports, the seeds of *Marrubium* species have oblong form while colors vary from brownish to greenish or dark brown [45,52]. Akgül et al. [45] grouped *Marrubium* species into three groups depending on the shape, size, and surface of the seeds. In this study, the ornamentation, the form, and the size of nutlets of *M. friwaldskyanum* were different from those in other *Marrubium* species and we cannot assign the seeds of this species into any of the groups described previously by Akgül et al. [45].

## 4. Materials and Methods

### 4.1. Collection of the Plant Material

An official permit (# 749/29.05.2018 of MOCB) for the collection of *Marrubium friwaldskyanum* Boiss. was obtained by the authors from the Bulgarian Ministry of the Environment and Water prior to the sampling of these endemic and protected plants.

Plant material of *M. friwaldskyanum* was collected in the fall of 2018 and 2019 from natural populations in Bulgaria, above the village Dobrostan, Rhodope Mountains (41°54′03.2″ N 024°54′16.4″ E; 1316 masl). The collected biomass samples were immediately transferred and then dried in an aerated shady place for a month until a constant weight was achieved before the essential oil was isolated. The collection of *M. friwaldskyanum* was deposited at the Herbarium of the Agricultural University, Plovdiv, Bulgaria (SOA) [55].

### 4.2. Essential Oil (EO) Extraction of Marrubium friwaldskyanum

In this study, the EO of *M. friwaldskyanum* was isolated via steam distillation and hydro-distillation in two separate experiments.

#### 4.2.1. Steam Distillation of the Essential Oil (EO)

A sample of fresh plant biomass consisting of stems and flowering parts was coarse ground using a Waring Commercial blender (Model CB15, McConnellsburg, PA, USA) and placed into a 2 L bioflask installed above the boiling flask. Water (1.5 L) was added to a two-neck boiling flask and distilled for 4 h (h). The distillation time was measured from the time the first drop of EO appeared in the glass separating funnel (the separator), and at the end of the 4 h period, the power was shut down, and the distillation discontinued. After the end of the distillation, 10 mL of pentane was used to dilute the EO and make sure all the EO was washed out from the side of the apparatus. The water was drained from the Florentine vessel and the oil/pentane mix was collected in a glass vial.

#### 4.2.2. Hydro-Distillation by Clevenger-Type Apparatus

Two separate experiments were conducted with hydro-distillation of *M. friwaldskyanum* (Table 5 and Table 6).

(a) Changes in the EO profile at different phenological stages (Table 5). Representative samples of *M. friwaldskyanum* were collected and hydrodistilled at three different phenological stages; (1) vegetative stage with plants at two-three pairs of leaves; (2) flower butt formation, at the pre-flowering stage; and (3) at full flowering stage. All samples were cut into 1.5–2.0 cm pieces just prior to distillation. The EO was isolated by hydrodistillation for 3 h in a Clevenger-type laboratory glass apparatus of the State Pharmacopoeia of the USSR [56] at the University of Food Technologies in Plovdiv. At the end of the distillation, because of relatively low EO yield, the EOs was washed out and dissolved in n-hexane to make sure all the EO was recovered. The EO samples were stored in tightly closed vials at 4 °C until analysis. The EO yields are reported on an absolute dry weight basis, as subsamples were taken from every batch and dried at 105 °C to a constant weight. The percentage yield of essential oil was calculated as per Equation (1).

Oil Yield % (𝑣/𝑤) = Volume of essential oil collected in mL/Weight of samples for extraction in grams ∗ 100.(1)

(b) The samples from the second experiment were extracted via hydrodistillation in Clevenger apparatus (Laborbio Ltd., Sofia, Bulgaria) for 2 h at the Research Institute for Roses and Medicinal Plants in Kazanluk, Bulgaria. The general method was described in the State Pharmacopoeia of the USSR [56]. The two experiments utilized slightly different modified extraction methods; however, we are not comparing the results from the two experiments.

### 4.3. Gas Chromatography Mass Spectrometry Flame Ionization Detection (GC-MS-FID) of Essential Oil (EO)

Due to the limited absolute amounts of EO obtained from the distillations, 10 mg of EO (or standard) was weighed directly into a 10 mL volumetric flask and brought to volume using CHCl_3_. A 1 mL aliquot of each EO sample or standard was placed by glass pipet into a GC vial for analysis.

*M. friwaldskyanum* EO samples were analyzed by GC–MS–FID on an Agilent (Santa Clara, CA, USA) 7890A GC system coupled to an Agilent 5975C inert XL MSD. Chemical standards and oils were analyzed using a DB-5 column (30 m × 0.25 mm fused silica capillary column, film thickness of 0.25 µm) operated using the following conditions: injector temperature of 240 °C; column temperature 60 to 240 °C at 3 °C/min, held at 240 °C for 5 min; carrier gas, He; injection volume, 1 µL (split ratio 25:1); MS mass range from 50 to 550 m/z; filament delay of 3.5 min; injection volume, 1 μL (split ratio 50:1); FID temperature was 300 °C. Post-column splitting was performed so that 50% of outlet flow proceeds to FID and 50% to mass spectrometry (MS) detection.

Compounds were identified by retention index (RI) and Kovats index (KI) analysis as previously described [57], direct comparison of MS and retention time to authentic standards, and/or comparison of mass spectra with those reported in the National Institute of Standards and Technology (NIST) mass spectra database. Commercial standards of (*E*)-caryophyllene, caryophyllene oxide, carvacrol, α-copaene, β-bourbonene, δ-cadinene, α-humulene, α-pinene, D-limonene, and Z-β-farnesene were obtained from Sigma-Aldrich (St. Louis, MO, USA). Spathulenol was available as an in-house standard previously isolated and identified. Commercial standards were purchased for all but four compounds (β-bourbonene, bicyclogermacrene, germacrene D, and τ-muurolol) allowing for unequivocal identification. Identification of β-bourbonene, bicyclogermacrene, germacrene D, and τ-muurolol was accomplished using RI and KI analysis as well as NIST library comparisons. Standards were injected and compared with retention time and mass spectra data of oil and used for identification where possible.

**Figure 7:**
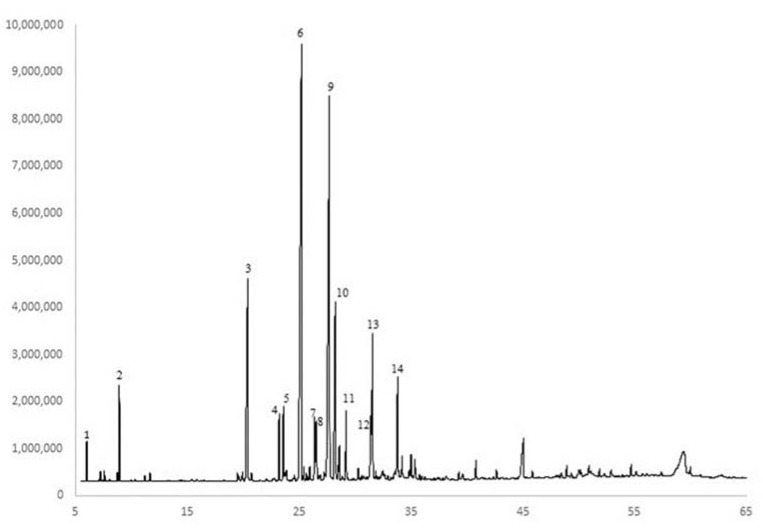
GC-FID chromatogram of a representative *M. friwaldskyanum* essential oil with major constituents labeled. 1—α-pinene; 2—D-limonene; 3—carvacrol; 4—α-copaene; 5—β-bourbonene; 6—*(E)*-caryophyllene; 7—α-humulene; 8—(Z)-β-farnesene; 9—germacrene D; 10—bicylcogermacrene; 11—δ-cadinene; 12—spathulenol; 13—caryophyllene oxide; 14—τ-muurolol.

Compounds were quantified by performing area percentage calculations based on the total combined FID area following the integration of major peaks. For example, the area for each reported peak was divided by the total integrated area from the FID chromatogram from all reported peaks and multiplied by 100 to arrive at a percentage. The percentage of a peak is a percentage relative to all other constituents integrated into the FID chromatogram (Figure 7).

### 4.4. Metabolomics Method

Above ground plant parts were ground using a coffee grinder. A 1 g aliquot of plant powder was extracted with 10 mL methanol, and the mixture was ultrasonicated for 30 min. After settling of the solids, 1 mL extract solution was transferred to a 1.5 mL tube and centrifuged at 14,000× *g* for 10 min. An aliquot of the supernatant (50 µL) was diluted 20 times with 50% aqueous methanol (950 µL). The mixture was vortexed for 10 s and centrifuged at 14,000× *g* for 10 min. The supernatant was transferred to a glass vial for LC-MS/MS analysis. Chromatographic separation was conducted using a Shimadzu Nexera UPLC system equipped with Inertsil Phenyl-3 column (150 × 4.6 mm, 5 µm). Mobile phase A was water with 0.1% formic acid, and mobile phase B was methanol with 0.1% formic acid. The gradient started with 5% B and was held for 1 min, followed by a 10 min linear gradient from 5% to 30%. The gradient was then stepped to 100% B at 23 min and held for 12 min and finally, stepped back to 5% B to equilibrate the column. The flow rate was 0.4 mL/min, and the column temperature was maintained at 45 °C. The UPLC system was connected to an AB Sciex Triple TOF 5600 mass spectrometer equipped with a TurboSpray electrospray ionization source operated in the negative ionization mode. The instrument was operated in the information-dependent acquisition (IDA) mode using a collision energy setting of 40 eV. Extract constituents were characterized by retention time, accurate mass, isotopic pattern, and MS/MS spectra and identified by LC-MS/MS comparison with 500 authentic standards (Natural Products Library, Enzo Life Sciences).

### 4.5. Scanning Electron Microscopy (SEM) Analysis

The scanning electron microscope (SEM) used in this investigation was an FEI Quanta 600 SEM at the Microscopy Facility at Oregon State University, United States. Sample preparation included placing small samples into a fixative, 1% paraformaldehyde and 2.5% glutaraldehyde in 0.1 M sodium cacodylate buffer with pH 7.4. The samples were soaked in fixative for 2 h followed by two rinses in 0.1 M Cacodylate buffer, 15 min each, and then by a dehydration series in acetone (10%, 30, 50, 70, 90, 95, 100–100%), 10–15 min each, followed by critical point drying (two ‘bomb flushes’ at chamber pressure to 5 °C, fill the chamber with CO_2_). The samples were left to vent for 5 min and then the procedure was repeated. The dry samples were mounted onto an aluminum SEM stub with double stick carbon tape. Samples were sputter-coated with a Cressington 108A sputter coater from Ted Pella with Au/Pd, 60/40 mix.

The nutlets (seeds) morphological description of species, the shape, as well as the structure of the surfaces were determined. In this case, the terminology and classification described by Barthlott and Ehler [9] were used. For pollen surface, we used the terminology and classification described by Punt et al. [58].

### 4.6. Embryological Analyses

Flowers and flower buds were used for embryological studies. The flowers and flower buds were collected from 30 individual plants from the population. The collected materials were fixed in a mixture of FAA (formalin: glacial acetic acid: 70% ethanol in a ratio of 5:5:90 parts, respectively). Then, the fixed plant material was treated according to the classical paraffin methods [59] to obtain permanent microscopic slides. The main embryological structures and processes in the male and female generative spheres were established via observations on an Olympus Light CX2 microscope (Olympus Corporation, Shinjuku, Tokyo, Japan). The microphotographs were prepared using an “Infinity lite” digital camera 1.4 Mpx.

#### 4.6.1. Pollen Viability

The pollen grains from 30 anthers from different individual plants were counted. The pollen viability was conducted using the Acetocarmine test [60]. Temporary slides were prepared after staining the pollen grains with a solution of 1% acetocarmine that resulted in pollen stained in red (viable, fertile) and unstained (nonviable, sterile). The mature pollen grains were counted (in the visual field at 100× magnification) using an Olympus Light CX2 microscope (Olympus Corporation, Shinjuku, Tokyo, Japan).

#### 4.6.2. Nutlets (Seed) Viability Testing

The *M. friwaldskyanum* seed viability was tested using the seed tetrazolium assay. Seeds were collected in July–August from different individual plants, and 100 seeds of *M. friwaldskyanum* were used for the tetrazolium test. Prior to the treatment with 1% solution of the tetrazolium chloride, the seeds were kept for 24 h in Petri dishes on wet filter paper at 25 °C temperature. The seeds were then incubated in 1% solution of 2,3,5-triphenyltetrazolium chloride following the procedure described by Peters [61]. At first, the tetrazolium solution was colorless. Later, its color changed from dark pink to red because of the action of hydrogen ions coming from the respiration process of the seeds. The viable embryos that showed a physiological activity (active respiration) turn red. The darker the red color, the greater the respiratory activity of the seeds.

#### 4.6.3. Testing of Nutlets (Seed) Germination at Different Light Regimes

The light intensity was 250 micromoles and the photoperiod was 16/8 h light/dark. The Petri dishes were placed at a 60 cm distance from the light sources. All Petri dishes were placed at a temperature of 23 °C during the day and 20 °C during the night. There were 50 seeds in each Petri dish. The experiment was performed with three replications for each variant. The seeds were kept moist by adding distilled water every second day. The seed germination energy was measured on the fifth day, and the total germination (according to the procedure described in BDS) was measured on the tenth day. The averages and standard deviation (SD) of the germination energy (%) and total germination (%) in triplicate were used for the Descriptive statistical analysis (Table 4). The wavelengths of the light colors are shown in Table 7.

### 4.7. Statistical Analyses

One-way ANOVA was conducted to determine the effect of (1) harvest stage (three levels: 2–3 pair of leaves, before flowering, and flowering) on the concentrations of 10 compounds: α-copaene, β-bourbonene, (*E*)-caryophyllene, α-humulene, germacrene D, bicyclogermacrene, δ-cadinene, spathulenol, caryophyllene oxide, and τ-muurolol; and (2) Grinding (4 levels: whole plant, ground/without water, fresh/ground with water, and ground/immediately extracted) on the concentrations of 14 compounds α-pinene, D-limonene, carvacrol, α-copaene, β-bourbonen (*E*)-caryophyllene, α-humulene, Z-β-farnesene, germacrene D, bicyclogermacrene, δ-cadinene, spathulenol, caryophyllene oxide, and τ-muurolol.

For all ANOVA, the validity of normal distribution and constant variance assumptions on the error terms was verified by examining the residuals as described in Montgomery [62]. The analysis was completed using the GLM procedure of SAS [63]. The effect of (1) harvest stage was significant only on α-copaene, (*E*)-caryophyllene, caryophyllene oxide; and τ-muurolol. The effect of (2) grinding was significant only on D-limonene and bicyclogermacrene. To differentiate the levels of the factors in terms of these compounds, multiple means comparison was conducted using Fisher’s LSD at the 5% level of significance.

## 5. Conclusions

This was the first phytochemical and embryological study on the Bulgarian endemic *M. friwaldskyanum* collected from wild populations in the Rhodope Mountains. The species mode of reproduction and reproductive capacity were established to define the character and state of the populations of this endemic species in connection with its preservation for the Bulgarian flora. The established peculiarities of reproductive biology provide a high reproductive capacity of populations, but its realization is limited from the environmental factors. Based on the attachment to certain environmental conditions of the species, it may be concluded that an appropriate strategy for its preservation is the monitoring and approaches for in situ conservation of the habitats.

Overall, the chemical composition of *M. friwaldskyanum* EO and some of the microscopy analyses distinguished this endemic species from other species in Marrubium. *Marrubium friwaldskyanum* may be promising to be developed as a new cash crop. It currently thrives on poor depleted soils high in the mountains. Therefore, one may assume it would provide comparable biomass and EO yields to the cultivated *M. vulgare* (horehound, currently among the top-selling herbal supplements in the U.S.) when grown as a cultivated crop on fertile agricultural soils under proper irrigation and fertilization regime. Indeed, this has been the case with a number of other wild medicinal plants when introduced in cultivation. Further research is needed to support these assumptions.

## Figures and Tables

**Table 1:** Mean concentration (%) of α-copaene, (*E*)-caryophyllene, caryophyllene oxide, and τ-muurolol obtained from the three levels of harvest stage where the effect of Harvest stage is significant.

Harvest Stage	α-Copaene	(*E*)-Caryophyllene	Caryophyllene Oxide	τ-Muurolol
2–3 pair of leaves	1.83 a	41.0 a	11.81 a	2.67 a
Before flowering	1.44 ab	39.3 a	9.98 ab	2.80 a
Flowering	1.26 b	30.8 b	6.35 b	1.32 b

Within each column, means sharing the same letter are not significantly different.

**Table 2:** Overall mean concentration (%) of the 6 compounds (β-bourbonene, α-humulene, germacrene D, bicyclogermacrene, δ-cadinene, and spathulenol) where there was no significant difference among the harvest stages.

Compound	Overall Mean Concentration (%)
β-bourbonene	1.10
α-humulene	2.83
germacrene D	23.30
bicyclogermacrene	2.85
δ-cadinene	1.07
spathulenol	2.83

**Table 3:** Mean concentration (%) of D-limonene and bicyclogermacrene obtained from the four levels of Grinding where the effect of Grinding is significant.

Grinding	D-Limonene	Bicyclogermacrene
	------ (%) is the GC-FID area ^1^ -----	
Whole plant	1.88 b	9.14 a
Grinded/without water	0.24 d	6.93 ab
Fresh/ground with water	1.08 c	3.55 b
Ground/immediately extracted	3.25 a	6.02 ab

Within each column, means sharing the same letter are not significantly different. ^1^ Mean concentration (%) is the GC-FID area percentage.

**Table 4:** Germination energy (%) and germination (%) of *M. friwaldskyanum*.

Variants	Germination Energy (%) ± SD	Germination (%) ± SD
Variant 1 (N)	24.0 ± 1.0	48.0 ± 2.0
Variant 2 (WR7:B1)	26.0 ± 1.0	56.0 ± 2.1
Variant 3 (WR4:B1)	26.0 ± 1.0	52.0 ± 1.0
Variant 4 (W)	27.0 ± 0.6	58.0 ± 2.0

Variant 1—natural daylight (N); variant 2—fluorescent white light with the addition of LED red and blue light in a ratio of 7:1 (WR7:B1); variant 3—fluorescent white light with the addition of red and blue light in a ratio of 4:1 (WR4:B1); variant 4—fluorescent white light (W); standard deviation—SD.

**Table 5:** Phenological stage, samples size (g), moisture (%), and oil yield (%) of *Marrubium friwaldskyanum* in Bulgaria.

Phenologycal Stage	Sample Size/g	Moisture Content (%)	Yield of EO (% *v*/*w*)	Yield of EO (%)
(1) 2–3 pairs of leaves	97.5	9.23	0.097	0.107
(2) pre-flowering flowering	86.5	9.45	0.074	0.082
(3) Flowering	159.5	62.19	0.012	0.034

The EO yield was recalculated and is reported in absolute dry-matter biomass.

**Table 6:** Results from the second experiment; treatments, samples size (g), and oil yield (%) of *Marrubium friwaldskyanum* in Bulgaria.

Treatments	Sample Size (g)	Water (mL)	Yield of EO, % Volume/Weight
(1) Fresh samples, whole plants, without grinding	200	1100	0.05
(2) Fresh, grinding without water	200	1100	0.03
(3) Fresh, ground with water	200	1400	0.04
(4) Fresh, ground with water, macerated with 0.1% Tween^®^ 20 for 12 h prior to the extraction	200	1400	0.04
(5) Fresh, ground with water and macerated with 0.1% Tween^®^ 20 and immediately extracted	200	1400	0.03

**Table 7:** LED bulb specifications.

Light Colour	Technical Specification			
	Power (W)	Tension (V)	Wavelength nm	Light Flux
Red/blue 7:1	200 W	220 V (85−265 V)	660 nm: 460 nm, (64 diode) of 3 W	50 sm–250 µmol/m^2^s
Red/blue 4:1	200 W	220 V (85–265 V)	660 nm: 460 nm, (64diode) of 3 W; 730 nm-2 pcs;	50 sm–250 µmol/m^2^s
White	200 W	220 V (85–265 V)	660 nm-26 pcs; 630 nm-16 pcs460 nm-9 pcs; 440 nm-6 pcs; 610 nm-2 pcs; 380 nm-1 pcs; 3500 k-2 pcs; (64 diode) of 3 W	50 sm–250 µmol/m^2^s
daylight				300 µmol/m^2^s–2000 µmol/m^2^s

## Data Availability

Data is contained within the article.

## Abbreviations

SEM	scanning electron microscopy
U.S.	United States
NPs	natural products
SOA	Herbarium of the Agricultural University, Plovdiv, Bulgaria
GC-MS-FID	gas chromatography mass spectrometry flame ionization detection
EO	essential oil
N	natural daylight
WR7:B1	fluorescent white light with the addition of LED red and blue light in a ratio of 7:1
WR4:B1	fluorescent white light with the addition of red and blue light in a ratio of 4:1
Wh	fluorescent white light
LED	Light-emitting diode
